# Implementation of the Multidisciplinary Guideline on Chronic Pain in Vulnerable Nursing Home Residents to Improve Recognition and Treatment: A Qualitative Process Evaluation

**DOI:** 10.3390/healthcare9070905

**Published:** 2021-07-16

**Authors:** Lizanne E. van den Akker, Margot W. M. de Waal, Paul J. E. M. Geels, Else Poot, Wilco P. Achterberg

**Affiliations:** 1 Department of Public Health and Primary Care, Leiden University Medical Center, Hippocratespad 21, 23333 ZD Leiden, The Netherlands; l.e.van_den_akker@lumc.nl (L.E.v.d.A.); w.p.achterberg@lumc.nl (W.P.A.); 2 Dutch Institute for Rational Use of Medicine (IVM), Churchilllaan 11, 3527 GV Utrecht, The Netherlands; p.geels@ivm.nl; 3 Dutch Association of Elderly Care Physicians (Verenso), Orteliuslaan 750, 3528 BB Utrecht, The Netherlands; epoot@verenso.nl

**Keywords:** pain, pain management, nursing home, guideline, implementation, pharmacotherapy audit meetings, qualitative research

## Abstract

The recognition and treatment of pain in nursing home residents presents challenges best addressed by a multidisciplinary approach. This approach is also recommended in the applicable Dutch guideline; however, translating guidelines into practical strategies is often difficult in nursing homes. Nevertheless, a better understanding of guideline implementation is key to improving the quality of care. Here we describe and qualitatively evaluate the implementation process of the multidisciplinary guideline ‘Recognition and treatment of chronic pain in vulnerable elderly’ in a Dutch nursing home. The researchers used interviews and document analyses to study the nursing home’s implementation of the guideline. The project team of the nursing home first filled out an implementation matrix to formulate goals based on preferred knowledge, attitudes, and behaviors for the defined target groups. Together with experts and organizations, pharmacotherapy audit meetings were organized, an expert pain team was appointed, a policy document and policy flowchart were prepared, and ‘anchor personnel’ were assigned to disseminate knowledge amongst professionals. Implementation was partially successful and resulted in a functioning pain team, a pain policy, the selection of preferred measurement instruments, and pain becoming a fixed topic during multidisciplinary meetings. Nevertheless, relatively few professionals were aware of the implementation process.

## 1. Introduction

While the normal aging process does not necessarily lead to pain, pain is common in older persons and is especially common among patients in nursing homes [1]. Although pain affects overall functioning in older adults, it often goes unrecognized by healthcare professionals, and research has shown both over- and undertreatment of pain [2,3].

The biopsychosocial model depicts pain as a synergy in which biological, psychological, social aspects, and health-related quality of life all interact and therefore cannot be perceived separately [4]. This suggests that the diagnosis and treatment of pain requires a multidisciplinary approach that combines pharmacological and non-pharmacological treatment strategies [3,5].

In addition to well-established pharmacological treatments, there are also non-pharmacologic treatments that can (partially) relieve pain in older adults [6] and allow lower doses of analgesics [5]. Although most care professionals will acknowledge the beneficial effects of non-pharmacological treatments and a multidisciplinary approach, these treatment options are rarely used in nursing homes, possibly due to the fact that many are not (yet) evidence based [7].

To address this issue in the Netherlands, a multidisciplinary guideline was developed in 2011 and revised in 2016: ‘Recognition and treatment of chronic pain in vulnerable elderly’ [8,9]. In the years after publication, it became apparent that few elderly care physicians in Dutch nursing homes had implemented the guideline in their daily practice. Previous studies have also shown that publication of a guideline does not guarantee use of that guideline in daily patient care [10,11,12,13]. A lack of specific knowledge, and attitudes and reactions to pain can all influence guideline adherence [14,15]. Furthermore, it is often difficult for nursing homes to translate guidelines into practical implementation strategies that meet the needs and limitations of the organization. Therefore, a project was designed to support nursing homes in developing their own implementation strategies based on the principles of effective healthcare improvement [16]. This was done together with two nursing homes that showed interest and were able to participate within the given timeline. The main goal of this project was for nursing homes to optimize care for their residents who suffered from pain with assistance from the Dutch Association of Elderly Care Physicians (Verenso) and the Dutch Institute for Rational Use of Medicine (IVM). Below, we describe and qualitatively evaluate the implementation of the multidisciplinary guideline in one nursing home.

## 2. Materials and Methods

### 2.1. Design and Setting

This qualitative study examined the process of implementing a multidisciplinary guideline in nursing homes, based on interviews and analysis of documentation from meetings. The Medical Ethical Committee of the Leiden University Medical Center (LUMC) approved the study (P18.076). In the Netherlands, nursing homes are funded by the government [17]. They employ elderly care physicians who work in multidisciplinary teams [18,19]. See also Supplementary File S1.

### 2.2. Sample

Two nursing homes participated in this study. Since implementation of the guideline involves many different disciplines working in a nursing home, the following target groups were distinguished: project leader, members of the pain team, pharmacist, nurses (registered and non-registered), paramedics, physicians, members of the client council, residents, and legal representatives. For the purposes of evaluation, healthcare professionals were asked to participate in interviews. The inclusion criterion for all participants was sufficient proficiency in the Dutch language and written informed consent. As sample size cannot be predetermined in qualitative research [20], recruitment of participants ended when no new insights (data saturation) emerged.

Before the study commenced, all healthcare professionals working on at least one of the three wards selected for implementation and residents (or their legal representatives) living on one of the selected wards received an information letter.

### 2.3. Procedure

The implementation process in the nursing home was predefined and divided into three phases—phase 1: preparation; phase 2: implementation of the guideline; phase 3: evaluation. Here we report on the situation before the implementation (T0) and on phase 1 and 2 of the implementation process.

Data on the implementation process were obtained from two sources: (1) semi-structured interviews with professionals, and (2) policy documents and records of meetings. The interviews took place at baseline (May and June 2018, T0) and at completion of the implementation project (July and August 2019, T1), but implementation documentation was gathered over the entire implementation period. Semi-structured interview guides were developed to explore the care professional’s perceptions of the process of implementation and the interviews were fine-tuned per profession (for the T0 and T1 interview guide for paramedics and nurses (registered and non-registered), see Supplementary File S2). The interviews were conducted face-to-face in the nursing home or by telephone, depending on the preference of the care professional, and lasted around 30 min. The interviewer made notes during and after each interview. The interviews were audio recorded and transcribed verbatim.

### 2.4. Materials

Development of materials by the Dutch Association of Elderly Care Physicians (Verenso) and the Dutch Institute for Rational Use of Medicine (IVM) took place alongside implementation in the nursing homes. During the preparation phase, IVM developed the pharmacotherapy audit meetings, the e-learning for nurses, and a flyer for patients (available online www.medicijngebruik.nl/over-ivm/onderwerpen-a-z/nieuws/4573/project-‘pijn-in-beeld-en-behandeld’ accessed on 15 July 2021). During the evaluation phase, Verenso developed an implementation manual and organized sounding board group meetings to evaluate all materials that ultimately will be included in the ‘toolbox’ alongside the guideline (available online www.verenso.nl/richtlijnen-en-praktijkvoering/richtlijnendatabase/pijn accessed on 15 July 2021).

### 2.5. Analysis

The transcribed interviews were content analyzed using ATLAS.ti version 7.5.10 (ATLAS.ti-Scientific Software Development GmbH, Berlin Germany), with open coding to address study objectives. The coding scheme was based on the topic questions. After open coding, broader themes and supporting quotes were identified [21].

## 3. Results

Two nursing homes completed implementation of the guideline. A nursing home organization in Vlaardingen with six locations (415 clients), the Netherlands, implemented the guideline in three selected wards (60 clients, mean age 80.8 years): geriatric rehabilitation (R) and somatic (S) and psychogeriatric (PG) care. Another nursing home in Breda, the Netherlands, also implemented the guideline on a somatic ward, a ward for psychogeriatric care, and a short stay ward. Due to the COVID-19 pandemic, only the first nursing home yielded a complete dataset and is described in this paper.

The opinions of professionals were collected through interviews. The estimated point of saturation was observed after 21 interviews during the T0 interviews, and after 18 interviews at T1. For more information on participant characteristics, see Table 1.

### 3.1. Opinions of Professionals before Implementation (T0)

Below, we describe their opinions regarding the original situation in themes.

- Need for implementation:

All interviewed professionals emphasized the importance of implementing the guideline, mentioning the positive influence on raising awareness of pain, and standardizing clinical procedures, which should eventually lead to better pain treatment and fewer patients with unnecessary pain. One registered nurse commented: ‘*The different kinds of pain don’t receive enough attention. We trust the physician’s medical approach, but pain can also highlight other problems. More education is paramount*’.

- Recognizing pain:

In general, all professionals considered themselves aware of pain and believed they could recognize almost all pain signals. Nevertheless, the professionals seemed open to further education concerning recognition of pain ‘*I can’t recognize what I’m unfamiliar with*’. Multiple problems were mentioned in relation to determining the presence of pain. ‘*Pain is a difficult symptom; residents might be in pain or might just be bored. A variety of factors can cause pain’*. *‘Pain is what a resident considers it to be*’. Furthermore, nurses mentioned frustration concerning the discrepancy between their observations and those of the physician. ‘*The physician often comes to the ward and sees a client for a short time, and may come to a different conclusion than the nurses. Some physicians take the nurse’s observations into account, while others draw conclusions solely based on their own observations*’. Most of the nurses interviewed were aware that they are ‘*The eyes and ears of the physician*’, but in some cases it was not clear how the nurses were expected to quantify and report pain to the physicians. Furthermore, nurses admitted that they do not persist in reporting pain in residents with chronic pain: ‘*That resident is just in pain*’.

- Measurement instruments:

All professionals acknowledged that pain measurement instruments should be used more often. The reasons mentioned for not using available instruments included a lack of knowledge regarding which instrument to use and when, a lack of evaluation of the results, and difficulties with entering results into the electronic patient information system. Physicians and nurses agreed that the use of measurement instruments would promote a structured approach and improve the speed with which treatments could begin, although one physiotherapist commented: ‘*I only have 30 min per client, which includes time for my administration. If I also have to use and report measurement instruments, I would not have any time left for my client*’.

- Non-pharmacological treatments:

Non-pharmacological treatments include physiotherapy, occupational therapy, and others, together with complementary treatments such as massage, aromatherapy, etc. The project leader admitted that complementary treatments in particular could be used more frequently. As one physician put it, ‘*Physicians like evidence… complementary treatments are a blind spot*’, even though all professionals acknowledged the potential positive influence of complementary treatments and that they are unlikely to do any harm. The project leader mentioned that it is often difficult to determine who is responsible for this type of treatment as, for example, a massage is no longer the responsibility of the physiotherapist. Furthermore, the project leader questioned the willingness of the nurses to add complementary treatments to their existing tasks.

- Pharmacological treatments:

Both nurses and physicians were generally satisfied with medical treatments and communication concerning these treatments. Some nurses mentioned difficulties with physicians: ‘*Some of them don’t listen to us, or it takes a while for them to start the right treatment*’. Physicians often begin treatments using low doses and during this dose-finding phase nurses must endure the sight of residents still in pain, which may lead them to conclude that the dose is incorrect. Furthermore, nurses mentioned that medical treatments are often not evaluated.

- Healthcare organization:

The nurses mentioned that they were not encouraged by their team leaders to be more aware of pain, probably due to the many other tasks facing team leaders. However, all nurses agreed that recognizing pain is part of their job. The nurse’s workload was also mentioned as an impediment to recognizing pain. Furthermore, due to the high turnover of professionals, it was harder to recognize pain in certain residents (it is easier to recognize that a resident is in pain when you know them well). A frequently mentioned barrier to recognizing and treating pain was the wide disparity in educational level between some professionals, which sometimes led to difficulties with interprofessional communication.

- Education:

The general view concerning pain education was universally positive. A few professionals were aware of the existence of a guideline, but most had never heard of it. Furthermore, actually reading the guideline was not feasible for most professionals due to a lack of time, and they were therefore primarily interested in clinical training or e-learning.

- Project management:

The project experienced some initial difficulties. Firstly, the timeline for the start of the project was too long, as the project leader mentioned: *‘Everybody involved was ready to begin but we still had to wait for the official start’*. Furthermore, nurses and paramedics felt that they did not receive enough information about the implementation of the guideline. Another issue was that the production of educational materials took longer than planned, resulting in the first meetings being less informative than they might otherwise have been. Nevertheless, the pain team members and physicians were very satisfied with the performance of the project leader, who effectively safeguarded the timeline and organized all meetings.

### 3.2. Phase 1: Preparation (6 Months)

Phase 1 started in February 2018. To summarize: the nursing home selected participating wards, all care professionals were informed, a pain team was installed, goals were formulated, and a meeting with the client council took place.

Potential pain team members were approached, and the six members finally selected consisted of a physiotherapist, an occupational therapist, a psychologist, a physician, and two registered nurses, with a speech therapist available on demand. All members participated as a project team and together filled out the implementation matrix provided by ZonMw [22]. This matrix distinguished specific goals, divided into *knowledge*, *attitudes* and *behaviors*, to be reached by the end of the implementation period for the defined target groups. The summarized main implementation goals of the matrix are displayed in Table 2. During this phase, meetings were organized with the relevant care professionals and the client council in order to keep everyone involved and up to date.

With help from the pharmacist, the pain team then catalogued prescribed medication and reached out to local general practitioners (GPs) to develop a standardized format for a transfer letter with the goal of improving communication between GPs and other care professionals concerning pain at discharge. In the following months, the project team focused on developing working procedures.

#### 3.2.1. Preparations by the Pain Team

In accordance with the guideline, the pain team selected preferred (self-reported and observational) pain measurement instruments and made a plan to implement structural pain registration. The members of the pain team received extra education about pain measurement instruments, the recognition of pain, current protocols, the perception of pain, and treatments. Furthermore, they had an opportunity to familiarize themselves with the expertise of the other pain team members. The pain team scheduled meetings every other week to create a flowchart, become familiar with the expertise of other pain team members, and to advise professionals regarding complex individual cases with chronic pain complaints. The anticipated benefit of the pain team was derived from the multidisciplinary approach to individual patients with pain.

The pain team instructed and assigned an anchoring role to one or two nurses per ward, and it was decided that they should complete the e-learning course ‘Care for nursing home residents with pain’ each year and score at least 7 out of 10 for the final test. The ‘anchors’ of the participating wards met each month to share knowledge about pain and to discuss their experiences of communication about pain. Furthermore, they were responsible for conveying information on pain to the residents/legal representatives by distributing the flyer ‘pain in vulnerable nursing home residents’ provided by Verenso and IVM [23]. The pain team’s registered nurse organized meetings with the ‘anchors’, provided clinical lessons, and coached the care professionals in how to use measurement instruments, formulate pain goals, and improve pain reports.

The pain team also assigned the nurses the role of encouraging the residents to discuss their pain. Furthermore, the nurses were expected to have an understanding of pain measurement instruments, to distract residents from their pain, to help them find a more comfortable position, to provide the correct medication, and to maintain contact with the family regarding pain. They were also responsible for setting pain goals and for monitoring and evaluating progress. Pain education was made available to the nurses.

#### 3.2.2. Working Procedures for the Pain Team

The project team formulated the pain team’s working procedures and created a flowchart for the nurses covering residents with known or suspected pain. This flowchart helps decide when to use which measurement instrument, whether the intervention is effective, and when it is time to ask a physician to assist the pain team. The project team agreed that a patient should meet the following criteria before assignment to a pain team: (1) presence of chronic pain (i.e., minimum of three months, no signs of tissue damage); (2) case exceeds the expertise of the ward; (3) case needs new or different insights; (4) pain seems inexplicable.

### 3.3. Phase 2: Implementation of the Guideline (8 Months)

A kick-off meeting marked the start of the implementation. Around 25 professionals were present: physicians, nurses, physiotherapists, and an occupational therapist. At the participating wards, information letters for patients were made available, and nurses were invited to follow the e-learning. Physicians, registered nurses, and a pharmacist were present at three pharmacotherapy audit meetings held at the start, during the course of, and at the end of the project. During these meetings, (non-)pharmacological treatment agreements were made and evaluated using data provided by a pharmacist (see Table 3). At the end of the implementation period, a functioning pain team was implemented, the pain policy was available for each professional, and pain was introduced as a fixed topic during the multidisciplinary meetings.

The project leader described progress made since the start of the project: ‘*There is greater awareness of pain, and paramedics are more open to providing complementary care’*. The pain team was generally enthusiastic about the project and their responsibilities, although the ‘anchors’ did face difficulties. All present at the kick-off meeting were enthusiastic about the meeting. Nevertheless, due to the high staff turnover and scheduling difficulties, most (registered) nurses and paramedics were not reached during the project: ‘*After the kick-off meeting, I didn’t receive any more information*’. Compared to the other wards, the somatic ward had the most stable group of professionals and a supportive team leader. The professionals who were aware of the project mentioned that it was often unclear what was expected of them. All professionals aware of the project mentioned they were happy with the project leader, who monitored the progress of the project.

Below, we further describe opinions of the professionals regarding the implementation process in themes:

- Pain diagnostics:

Pain team members and (registered) nurses acquainted with the project reported greater awareness of pain in residents. This was not the case for professionals who were unaware of the pain project, who reported that they had not noticed any differences since the start of the project. The members of the pain team also felt more confident after the implementation of the guideline in terms of recognizing pain.

- Measurement instruments:

During the implementation period, the nursing home introduced a new patient registration software system that included standard Numeric Rating Scales (NRS) forms and ‘Pain Assessment Checklist for Seniors with Limited Ability to Communicate’ (PACSLAC-D) forms [24]. Furthermore, professionals were now able to report on (pain) goals in the new registration software. Although it took some time for those involved to become accustomed to the new system, during the T1 measurements, three of the registered nurses instructed colleagues on how to work with the new system. The interviewed professionals who were aware of the project used measurement instruments more often.

- Non-pharmacological treatments:

The professionals did not notice any differences in how often non-pharmacological treatments were used. A physician commented: ‘We are trying to change medical culture to promote ‘non-evidence based’ treatments; this process takes time’. However, the somatic ward did assemble a ‘pain box’ containing items related to complementary treatments, such as a heating pad, massage oil, and aromatherapy equipment.

- Pharmacological treatments:

One physician mentioned that it was difficult to notify patients regarding how and why their pain medication needed to be reduced, especially during busy periods. How the medical treatments prescribed by the physicians were influenced by the pharmacotherapy audit meetings, as they were now more aware of problems and wished to quickly reduce pain medication and to plan evaluation moments. Overall, the professionals were satisfied with the pharmacological treatment of pain, but two nurses mentioned that in some cases pain medication should be started more quickly. The physicians and pharmacist were satisfied with the pharmacotherapy audit meetings.

**Figure 1:**
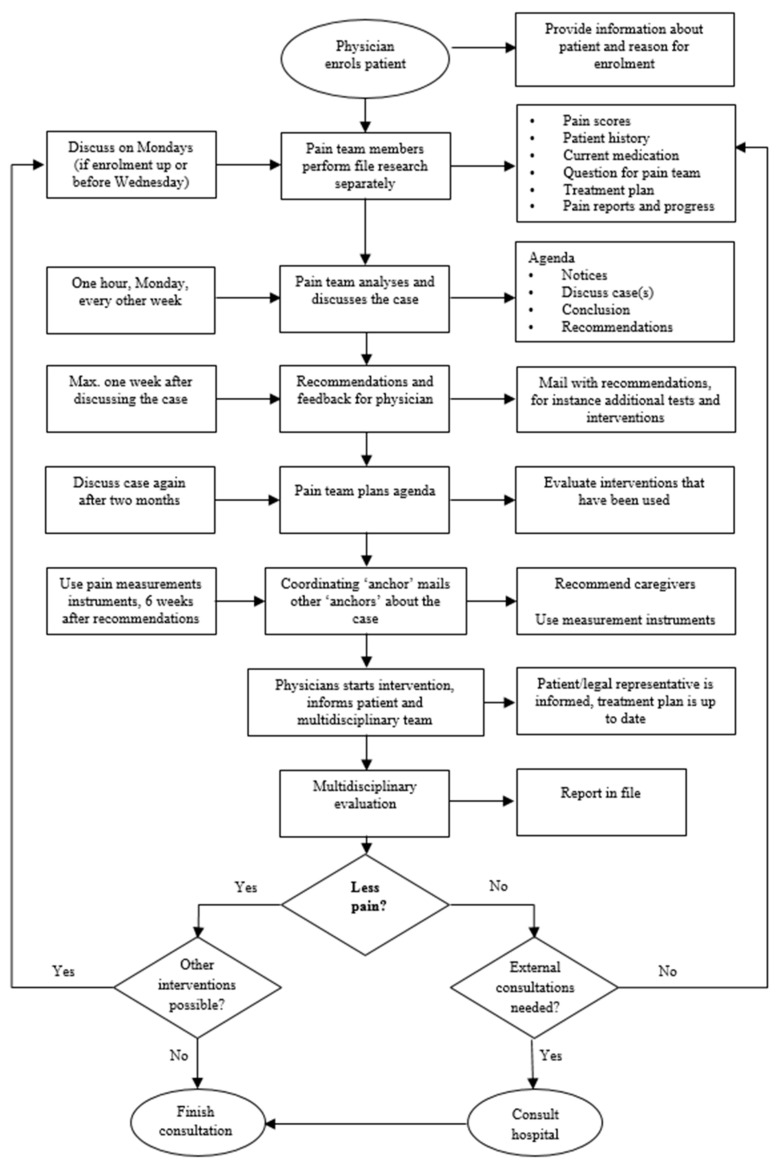
Work flowchart used by the pain team.

- Healthcare organization pain team:

The pain team was also satisfied with the policy document and flowchart (see Figure 1) they created. The multidisciplinary aspect of the team provided considerable positive motivation: ‘We all view the problem from the perspective of our own expertise and thus complement each other’. A speech therapist was available when necessary. To ensure that the only cases provided fulfilled the criteria, the pain team decided that cases could only come from a physician. Furthermore, the pain team made pain a standard subject during multidisciplinary meetings.

The pain team discussed three cases over a period of 8 months. One member commented: ‘I think we only received 3 cases because physicians consider it is a sign of weakness to consult others, and they want to solve problems themselves’. The pain team advised non-pharmacological treatments for the three cases they received, and during the pain team evaluation, pain was found to be more bearable for two of these clients. In the other case, the family preferred a pharmacological treatment, which was provided.

- Education:

While the professionals were satisfied with the e-learning provided, most stated that they preferred lessons based on a (fictitious) case and mentioned that they already had a large amount of e-learning to complete. A team leader mentioned that e-learning should be provided every other year to ensure that new professionals were also up to date. The members of the pain team were familiar with and understood the workings of the guideline. However, most physicians and paramedics interviewed were unfamiliar with the guideline and mentioned that their familiarity with it had not increased since the start of the project.

- Advice for future implementation in other nursing homes:

The project leader mentioned the considerable amount of time required to implement the guideline, and although an eight-month period was initially allocated, in hindsight, this should have been at least a year. Furthermore, the necessary materials were not yet ready when the nursing home began implementation, which should be a requirement before kick-off: ‘The first meeting should be informative in order to create general enthusiasm’. The project leader also commented ‘The preparation phase needs to be kept short, otherwise enthusiasm can easily evaporate’. Interestingly, the most stable ward in terms of professional turnover also provided the most input (e.g., the painbox), suggesting that a stable ward may be an important prerequisite in implementing a guideline. In terms of improvements, more updates and information were mentioned, and others mentioned that newsletters would have been welcome.

- Sustaining implementation over the longer term:

During the T1 interviews, it became clear that not all initiatives had been implemented. The nursing home was working on the systematic use of patient registration software for pain reporting, implementing ‘pain boxes’ with complementary treatments in all wards, and had planned clinical lessons for the professionals and ‘anchors’. Furthermore, there were plans to implement the guideline in all wards, to make e-learning available to all care professionals and to arrange financial compensation for the ‘anchors’. In this manner, the nursing home hoped to successfully sustain implementation of the guideline.

## 4. Discussion

This study explored the implementation process of the multidisciplinary guideline on the recognition and treatment of chronic pain in vulnerable elderly. A Dutch nursing home assembled a pain team, assigned anchor roles, created a flowchart-based pain policy, designated preferred measurement instruments, and applied an implementation toolbox. Professionals of participating wards confirmed that measurement instruments and non-pharmacological treatments were used more frequently and that pain became a permanent topic during multidisciplinary meetings. The project leader and pain team were highly motivated and enthusiastic concerning the project, although they realized that full implementation would be a major task.

The greatest advances were noted on the somatic ward, and this ward had the most involved anchor personnel. For instance, ward personnel had developed a practical pain box that included materials to support complementary care. Compared to the other wards, the somatic ward had the most stable group of professionals and a supportive team leader. This accords with previous research, which has shown that a supportive culture, a shared focus to change, and a motivational leader are key factors in the implementation and sustainability of guidelines [25,26,27]. A recent study found that use of pain management champions can increase self-efficacy and induce behavior change [28].

In this publication, we focused agreements (on processes outcomes) that were formulated (based on the goals) by the nursing home and evaluated in the pharmacotherapy audit meetings. For this, the pharmacist provided feedback on analgesic prescriptions to individual physicians. Unfortunately, these data could not be translated to meaningful project outcomes. This problem is recognized by others, and in line with one of the lessons from Quality Improvement Collaboratives that one should pay special attention to the collecting, processing, and interpreting of data [27]. However, we plan to analyze and discuss documentations in patient files and questionnaires in a succeeding publication. The implementation process was not entirely completed before the T1 measurements took place, and the most frequently mentioned explanation was that an implementation period of eight months was too short. Implementing a guideline requires a great deal of time and effort and involves many and varied aspects such as communicating with and instructing multiple target groups and professionals, changing standard routines, embedding new processes in usual care, and effectively creating ‘new’ functions for pain team members and anchor personnel. During the T1 measurements, the nursing home was still working on several initiatives concerning implementation of the guideline, such as broadening implementation to all wards, implementing pain toolboxes for complementary care, making e-learning available to all (registered) nurses, and informing professionals about the new electronic patient information system. Due to this ongoing process, it is likely that we did not fully capture the effect of implementation at T1. A further complication is that multiple projects and processes are always underway in a nursing home, so it is difficult to focus on a single project. Finding the necessary balance for an optimal timeline is difficult because a shorter implementation period would make it harder to reach all professionals, while a longer period might lead to a dilution of effort as other projects consume time and energy. After completion of the implementation period, few (registered) nurses and paramedics were aware of the implementation project, as most did not receive additional information after the kick-off meeting. One explanation was poor communication due to the high turnover rate of anchor personnel, and another was difficulty clearing (registered) nurse schedules to ensure that they were available for instruction and meetings. Previous research has also shown that a high turnover rate of care professionals negatively influences guideline adherence in nursing homes and that it can lead to a fluctuating focus on pain and weaken resident–professional relationships [29].

The pain team received only three cases for consultation in eight months, whereas the members of the pain team had expected that more cases would be referred. One explanation for the lack of referrals was the ‘physician culture’: a reluctance to consult other professionals. However, others stated that there were no other clients with complex chronic pain. Only physicians could refer a client to the pain team, but future nursing homes could also examine outcomes resulting from (registered) nurses referring clients to the pain team (while notifying physicians). Furthermore, in this project, the pain team was introduced as an ‘expert team’. The other nursing home decided to introduce the pain team as a multidisciplinary consultation team, which it in fact is, perhaps lowering the threshold for physicians to consult the pain team. In addition, they included anchor personnel in the pain team as a way to increase involvement with the project, spread knowledge concerning the recognition and treatment of pain, and to leverage the expertise of the pain team. However, it is important to keep in mind that each nursing home has an individual culture, working methods, hierarchies, etc., therefore each nursing home will need to tailor implementation strategies to the culture and context of that particular nursing home. An essential prerequisite for successful implementation is a nursing home culture that stimulates working according to guidelines [25,30].

In this project, the nursing home selected preferred pain measurement instruments, whereas previous research has shown that assigning measurement instruments is not enough [31]; paramedics and (registered) nurses also need to feel confident about using these instruments and should know how to register results in the electronic patient information system and effectively communicate this to the team. A complication in this case was that the nursing home introduced a new electronic patient information system during the project. An advantage of the new system was that it eased the registration of pain scores and the determination and reporting of pain goals. However, informing and awakening the interest of (registered) nurses, paramedics, and clients/legal representatives in a new registration system takes time. During the T1 measurements, three registered nurses were assigned to inform and instruct all users regarding the registration system.

We found that the attitude of personnel was not an obstacle to implementation, as all professionals were highly motivated to implement the guideline. Unfortunately, awareness of the project was not widely shared, since information did not reach most of the (registered) nurses. This is in line with a systematic review concerning standards for infection prevention and control among nurses, where most participants of the studies had adequate knowledge and a positive attitude, but (when measured) average to poor level of practice [32]. Recommendations from included studies comprised providing periodic training via conferences and relevant practical courses, providing training at the start of employment, and combining up-to-date theoretical and practical problems. Research on improvement of pain management also shows, that training an entire multidisciplinary team can support implementation, as it facilitates interdisciplinary learning, collaboration, and communication [26]. Training should address barriers and facilitators on domains of capability, opportunity, and motivation. To establish new routines, a more complex approach is necessary to influence motivation and ultimately change behavior [33].

Some suggestions for improvements to future implementation research: Firstly, implementation materials and information about the importance of implementing the guideline should be present at the kick-off meeting. Secondly, everyone involved needs to be informed about their role and updated about any progress made (for example via newsletters). This way, everyone feels more involved and motivated to implement the guideline. Thirdly, during the preparation phase, timing of communication to stakeholders is important, since enthusiasm declines if staff are eager to start but have to wait for an official kick-off meeting. Fourthly, since implementing a guideline is very time-consuming the implementation phase requires more than 8 months. Finally, in order to successfully implement a guideline, anchor personnel and members of the pain team should be stimulated to attend meetings and actively encouraged to participate in implementation.

## 5. Conclusions

In conclusion, implementing a multidisciplinary guideline is not to be taken lightly. We have described the various actions that were taken in a nursing home to improve pain management. This project has yielded an implementation toolbox useful to other nursing homes wishing to implement the guideline, including information on pharmacotherapy audit meetings, an implementation manual, e-learning for nurses, and a flyer for patients. However, evaluation also showed that implementation did not reach all healthcare professionals and that securing new routines will need ongoing attention. Suggestions for improvement include (timing of) communication to stakeholders, with clear materials and descriptions of roles, and motivational support.

We hope that the toolbox, together with process knowledge gained during this project, will ease future implementation and as such improve the care for nursing home residents with pain.

## Figures and Tables

**Table 1:** Participant characteristics.

	T0 Baseline	T1 end of Implementation Phase
Total participants, *n*	21	18
Sex, *n* (%)		
- Male	4 (19)	2 (11)
- Female	17 (81)	16 (89)
Role or profession, *n* (%)		
- Project leader (policy advisor)	1 (5)	1 (6)
- Pain team (nurse, physiotherapist, occupational therapist)	3 (14)	3 (6)
- Pharmacist	1 (5)	1 (6)
- Nurses and paramedics	13 (62)	11 (61)
- Physicians	3 (14)	2 (11)

**Table 2:** Summary of implementation goals.

1	Structural use of pain measurement and observation tools	Behavior
2	A functioning pain team that has access to necessary means	Behavior
3	An educated pain team that is familiar with the multidisciplinary guideline ‘Recognition and treatment of chronic pain in vulnerable elderly’ by Verenso * [8]	Knowledge
4	Overview of prescriptions and review of pain medication available through the pharmacist	Behavior
5	Pharmacotherapy audit meetings supported by data provided by the pharmacist	Behavior
6	Nurses and physicians operate according to the guideline	Behavior
7	Nurses receive extra education through the e-learning module provided by IVM *	Knowledge
8	Pain is integrated as a care goal for all care professionals	Behavior
9	Paramedics receive extra pain education	Knowledge
10	Paramedics help other disciplines to find non-pharmacological treatments for pain	Attitude and behavior
11	Paramedics accurately and fully report pain in patient files and letters of transfer to ‘first-line’ care	Behavior
12	Residents and family have been informed with available information	Knowledge
13	Residents and family know who they can contact for more information on pain and (non-)pharmacological treatment options	Knowledge
14	Residents/legal representatives have been informed about the care and treatment plans	Knowledge
15	The client council is informed and updated about the progress of guideline implementation	Knowledge

* Verenso = Dutch Association for Elderly Care Physicians; IVM = Dutch Institute for Rational Use of Medicine.

**Table 3:** Summary of pharmacotherapy audit meetings.

Meeting 1			Meeting 2		Meeting 3	
Agreement	Goal	Action	1st Evaluation	Action	2nd Evaluation	Action
1. Each NH resident will be systematically checked for pain complaints	After three months, 75% of vulnerable elderly have received a systematic check for pain complaints	Use of pain measurement instruments on pilot wards; role for ‘anchors’	75% is too high, goal is not reached; systematic check should be part of MDM and treatment plan	Handover to the attendings’ meetings; anchor personnel and physicians instruct other professionals to use instruments and document the outcomes	More systematic focus on pain is needed	Further promote and facilitate role of physicians and ‘anchor personnel’
2. Determine available and feasible non-pharmacological treatments for pain	At three months, there is a list of non-pharmacological treatments that physicians can use	The pain team and physicians create a list with available and feasible non-pharmacological treatments for pain	The pain team created a list, and non-pharmacological treatments are discussed during the attendings’ meetings. The pain policy is adjusted, with a description for each discipline	Agreement 2 will be maintained; request to share experiences with pain team; promote use of patient information flyer on pain	Only few cases are discussed with pain team	Promote use of flowchart to consult pain team; involve not only wards of pilot, but all wards of NH
3. Chronic paracetamol users receive 2.5 g instead of 4.0 g/day	After three months, 95% of chronic users receive max 2.5 g/day. No paracetamol as needed (prn) on the psychogeriatric ward	Pharmacist makes list of all chronic users; to be evaluated by physicians, max 2.5 g. Physicians give all new paracetamol users 4.0 g/day for four weeks, after which an evaluation is planned. Check whether long-lasting opiates are prescribed instead. The pharmacist makes a list for paracetamol prn users. New dose is 2.5 g/day; 3 × 500 mg and 1 × 1000 mg at night	Some stress mentioned, differences in opinions between wards, and between patients. Overall, prescriptions conform with guideline. Large variation in prescriptions; work toward standard prescriptions, and define reason for prescription	Agreement 3 will be maintained but nuanced: evaluation moment after 2 to 4 weeks with note in file; when paracetamol and opiates are combined, note order of tapering off medication; consider laxatives with opiates	Only small improvements in cut down of doses of paracetamol to 2.5 or max 3.0 g/day. Cut down is difficult, e.g., when pain is chronic. Cut down of opiates is preferred. Laxatives are more consistently prescribed with opiates	Write the indication in the comments field; this helps to consciously decrease or stop pain medication
4. Physicians no longer prescribe NSAIDs for vulnerable elderly without arthritis	After 1 month, vulnerable elderly without arthritis no longer receive NSAIDs	The pharmacist requires an explanation for NSAID prescriptions. The reasons are registered in the patient file and evaluations are planned	NSAIDs were not often prescribed. The agreement to stop prescription of NSAIDs is difficult for residents. Explaining side effects might prove beneficial	Agreement 4 will be maintained. Difficult to get freelancers † on board	No differences, despite notices from pharmacist. NSAID is needed, for e.g., for colic pain (prn). Physicians state: NH residents do not want to stop NSAIDs	Ending NSAID use is not feasible, write down indication and moment of evaluation

† The group of physicians consisted of physicians employed by the nursing home and self-employed physicians (freelancers). NH = nursing home; MDM = multidisciplinary team meeting; prn ‘pro re nata’ = when needed/required.

## Data Availability

The data presented in this study are available on request from the corresponding author. The data are not publicly available due to the confidentiality of involved professionals and organizations. We will be able to provide a detailed metadata description of our datasets, including machine-readable Dublin Core files. We will specify how researchers interested in our data can contact us via our data access committee. The data access committee is responsible for reviewing applications and for data access in agreement with the given informed consents.

## Supplementary Materials

The following are available online at https://www.mdpi.com/article/10.3390/healthcare9070905/s1, File S1: Nursing homes in the Netherlands, File S2: Semi-structured interview guide for paramedics and (registered) nurses.
