# Supplementary material for: Implementation of the Multidisciplinary Guideline on Chronic Pain in Vulnerable Nursing Home Residents to Improve Recognition and Treatment: A Qualitative Process Evaluation
Source: Healthcare (Basel). 2021 Jul 16;9(7):905. doi: 10.3390/healthcare9070905 (PMC8305707; doi:10.3390/healthcare9070905)
Supplement: Supplementary file 1 [file healthcare-09-00905-s001.zip › supliment 1.pdf]

Supplementary Material 1

Nursing homes in the Netherlands

In the Netherlands a nursing home (NH) is an institution which provides temporary or permanent multidisciplinary treatment, guidance and support, and nursing care for older patients with long-term, complex health problems, expressed primarily in functional disorders and disabilities. All Dutch NHs are funded by the government, under the 1968 Exceptional Medical Expenses Act – "Algemene Wet Bijzondere Ziektekosten" (AWBZ). The AWBZ stimulated the development from 60 NHs in 1964 to approximately 400 with a total of 65,000 beds in 2015. [1]

Dutch NHs have several functions. They represent a home for their residents while also providing nursing care, supervision, diagnostics, treatment and reactivation & rehabilitation. Of all patients newly admitted to a NH, 36% of the somatic patients stay longer than 6 months; while 66% of the psychogeriatric patients stay longer than 6 months. In total 33% are eventually discharged home, or moved to a residential care setting. Of patients admitted for geriatric rehabilitation about 80% returns to their own home. As an alternative to regular NH care, many awaiting patients receive 'NH care' in day care, residential homes, or in their own home. [1]

In the Netherlands NH physicians are named elderly care physician (ECP): a medical practitioner who has specialized as a primary care expert in geriatric medicine and qualified as a basic specialist with expertise in geriatric medicine. [2]

Institutions employ ECPs working in a multidisciplinary team. A multidisciplinary team consists of nurses, physiotherapists, occupational therapists, speech therapists, psychologists, social workers and pastoral workers and of course of ECPs. Nursing care is delivered by registered nurses, certified nursing assistants and nursing aides. [3]

References

1. Achterberg W, Caljouw M, Husebo BS. Towards academic nursing home medicine: a Dutch example for Norway? Omsorg 2015;1:70-75.
2. Koopmans RT, Lavrijsen JC, Hoek JF, Went PB, Schols JM. Dutch elderly care physician: a new generation of nursing home physician specialists. J Am Geriatr Soc 2010 Sep;58(9):1807-9.
3. Schols JMGA. Nursing home medicine in the Netherlands. Eur J Gen Pract 2005;11(3-4):141-3.
