# Supplementary material for: Implementation of the Multidisciplinary Guideline on Chronic Pain in Vulnerable Nursing Home Residents to Improve Recognition and Treatment: A Qualitative Process Evaluation
Source: Healthcare (Basel). 2021 Jul 16;9(7):905. doi: 10.3390/healthcare9070905 (PMC8305707; doi:10.3390/healthcare9070905)
Supplement: Supplementary file 1 [file healthcare-09-00905-s001.zip › supliment 2.pdf]

Supplementary Material 2

Semi-structured interview guide for paramedics and (registered) nurses, used to explore the care professionals perceptions on the process of implementation.

Baseline

Topic questions:

- What is your opinion about the implementation of the guideline ‘pain in vulnerable elderly’?
- Which information did you receive about the forthcoming implementation?
- Are you familiar with the guideline?
- Are you attentive regarding pain in residents?
- Do you know how you can recognize pain in nursing home residents?
- Are you encouraged by co-workers to be attentive regarding pain?
- Are you satisfied with the current procedures to recognize pain?
- Are you satisfied with the current procedures to treat pain?
- When you have questions about a pain treatment in a resident, can you discuss this with a physician?
- Do you believe that it is feasible to structure the use of pain measurement instruments?
- Is your understanding of pain sufficient to implement the guideline in your daily routine?

After the implementation period

Topic questions:

- Which steps were taken to implement the guideline?
- Which steps did you undertake yourself?
- Are you satisfied with the way these steps were taken?
- Which problems did you encounter?
- Are you satisfied with communication regarding the implementation of the guideline?
- Was your role in the implementation process clear to you?
- Do you believe that your understanding of pain was sufficient during the implementation period?
- Did you read the (summary of the) guideline?
- Are you more attentive regarding pain in residents compared to before implementation of the guideline?
- Are you now more encouraged by co-workers to be attentive regarding pain?
- Do you notice differences in recognizing pain since the implementation of the guideline?
- Do you notice differences in the treatment of pain since the implementation of the guideline?
- Do you notice differences in the use of pain measurement instruments since the implementation of the guideline?
- Was your role clear during the implementation period?
- What is your advice for future nursing homes planning to implement the guideline?
